# Supplementary figures and images for: Impact of cilostazol on clinical outcomes in lower extremity arterial disease patients after angioplasty: A real-world analysis
Source: PLoS One. 2025 Aug 21;20(8):e0330434. doi: 10.1371/journal.pone.0330434 (PMC12370041; doi:10.1371/journal.pone.0330434)

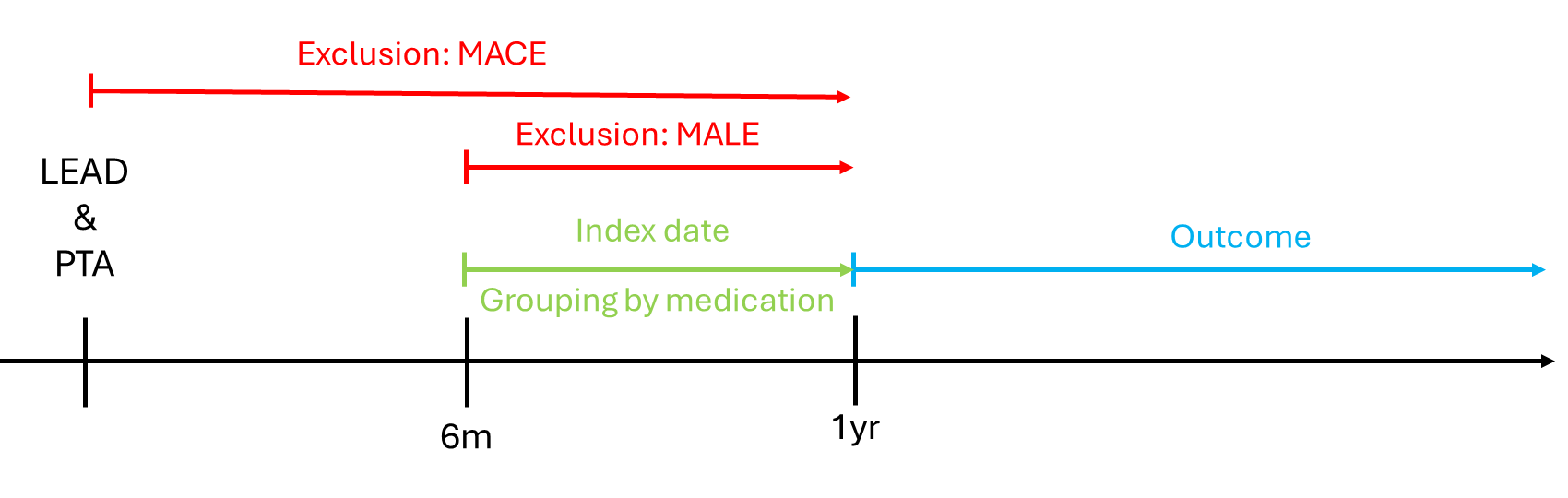

Supplement: Supplemental Fig 1 — (TIF) [file pone.0330434.s001.tif]

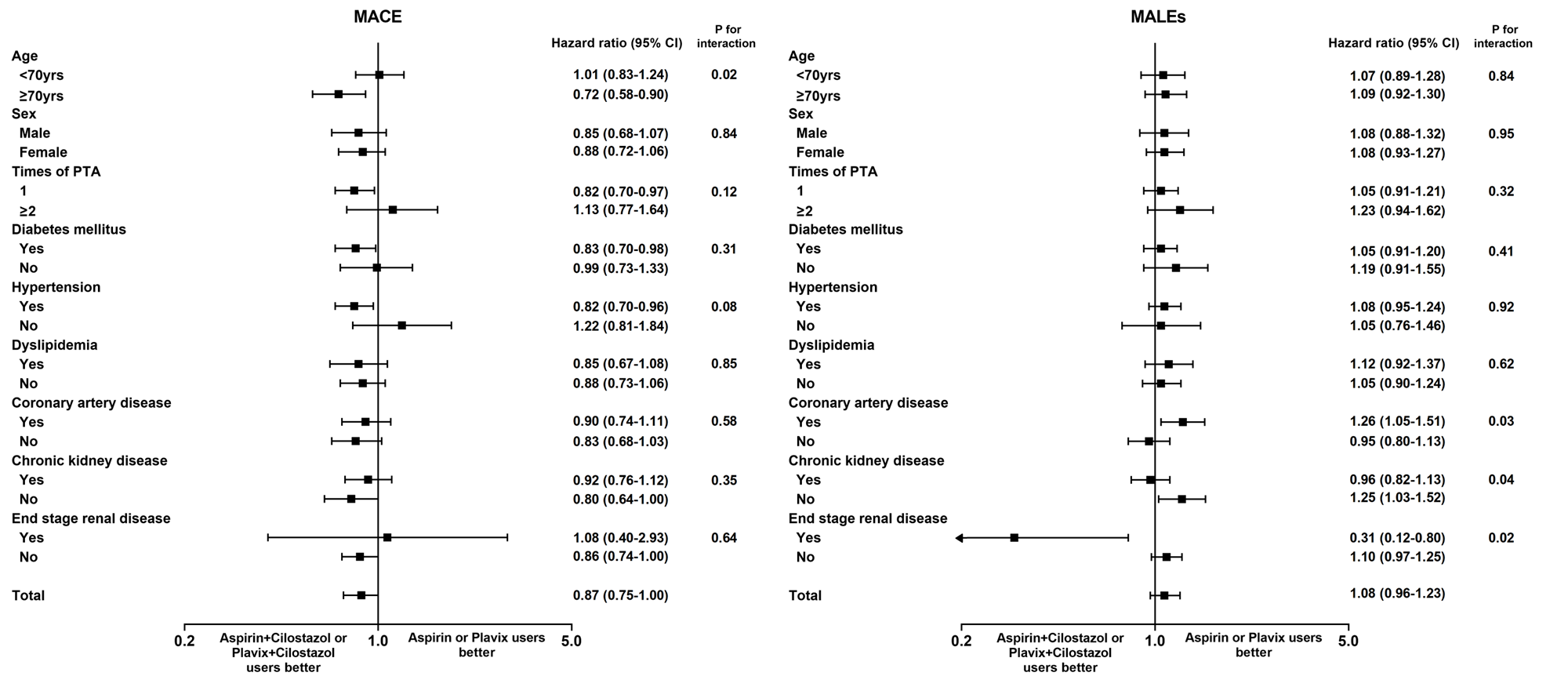

Supplement: Supplemental Fig 2 — (TIF) [file pone.0330434.s002.tif]
