# Supplementary material for: Impact of cilostazol on clinical outcomes in lower extremity arterial disease patients after angioplasty: A real-world analysis
Source: PLoS One. 2025 Aug 21;20(8):e0330434. doi: 10.1371/journal.pone.0330434 (PMC12370041; doi:10.1371/journal.pone.0330434)
Supplement: S1 Table — (DOCX) [file pone.0330434.s003.docx]

Supplemental table 1. ICD-9 and ICD-10 codes

| Disease | ICD-9 Codes | ICD-10 Codes |
| --- | --- | --- |
| Peripheral artery disease | 443.9, 440.2, 440.3, 440.4 | I65.9, I63.00, I63.10, I63.20, I63.29, I73.9, I70.2, I70.3, I70.4, I70.5, I70.6, I70.7, I70.8 |
| Comorbidities | | |
| Hypertension | 401,402,403,404,405 | I10-I16 |
| Diabetes mellitus | 250 | E10, E11 |
| Hyperlipidemia | 272 | E78 |
| Chronic kidney disease | 580-589 | I12, I13, N00, N01, N02, N03, N04, N05, N07, N11, N14, N17, N18, N19, Q61 |
| ESRD | 585.6 | N18.6; Z99.2 |
| Congestive heart failure | 428 | I5020, I5021, I5022, I5023, I5030  I5031, I5032, I5033, I5040, I5041  I5042, I5043, I509, I501 |
| Atrial fibrillation | 427.31 | I48.0, I48.1, I48.2, I48.9 |
| Coronary artery disease | 410,411,412,413,414 | I21-I25 |
| Ischemic stroke | 433, 434 | I63 |
| Transient ischemic attack | 435.9 | G45.9 |
| Hemorrhagic stroke | 430-432 | I61 |
| Outcome | | |
| MACE |  |  |
| Ischemic stroke | 433, 434 | I63 |
| Transient ischemic attack | 435.9 | G45.9 |
| Hemorrhagic stroke | 430-432 | I61 |
| Non-fatal MI | 410 | I21,I22,I23 |
| Safety outcome |  |  |
| Hemorrhagic stroke | 430-432 | I61 |
| Gastrointestinal bleeding | 456.0, 456.2, 455.2, 455.5, 455.8, 530.7, 530.82, 531.0–531.6, 532.0– 532.6, 533.0–533.6, 534.0–534.6, 535.0– 535.6 537.83, 562.02, 562.03, 562.12 562.13 568.81, 569.3, 569.85, 578.0, 578.1, 578.9 | K22.6, K25.0, K25.2, K25.4, K25.6, K26.0, K26.2, K26.4, K26.6, K27.0, K27.2, K27.4, K27.6, K28.0, K28.2, K28.4, K28.6, K29.0, K62.5, K92.0, K92.1, K92.2, I85.01 |
| Other critical sites bleeding | 423,0, 459.0, 568.81, 593.81, 599.7, 623.8, 626.32, 626.6, 719.1, 784.7, 784.8, 786.3, 519.09, 785.59, 958.4, 860.2, 860.3, 861.21, 861.31, 862.0, 862.1, 862.21, 862.31, 862.22, 901.0, 901.40, 901.41, 901.42, 864.00, 864.10, 965.00, 902.9, 865.10 | D62, J942, H113, H356, H431, N02, N95, R04, R31, R58, I71, K66.1, J95.01, R57.1, J94.2, T79.4XXA, S27.1XXA, S27.329A, S26.01XA, S27.809A, S27.409A, S27.819A, S25.00XA, S25.409A, S36.119A, S36.00XA, S35.90XA, S36.90XA, S35.00XA, S35.10XA, S35.299A, S35.403A, S35.513A, S31.001A, T79.4XXA, S37.009A, S37.90XA, S48.019A, S48.919A, S58.019A, S58.919A, S68.419A, S78.019A, S78.919A, S88.019A, S88.119A, S98.019A |
| Procedures | | |
| PTA | 38.08, 38.18, 38.38, 38.48, 38.58, 35.68, 38.88, 39.50, 39.7, 39.90, 39.25, 39.26, 39.29 | 041, 045, 047, 049, 04B, 04C,  04H, 04J, 04L, 04N, 04P, 04Q, 04R, 04S, 04U, 04V, 04W |
| Amputation | 84 | 0Y6 |
