# Supplementary material for: Impact of cilostazol on clinical outcomes in lower extremity arterial disease patients after angioplasty: A real-world analysis
Source: PLoS One. 2025 Aug 21;20(8):e0330434. doi: 10.1371/journal.pone.0330434 (PMC12370041; doi:10.1371/journal.pone.0330434)
Supplement: Attachment — Submitted filename: PONE-D-25-23547-RB.pdf [file pone.0330434.s004.pdf]

PLOS ONE

Impact of Cilostazol on Clinical Outcomes in Lower Extremity Arterial Disease Patients After Angioplasty: A Real-World Analysis

--Manuscript Draft--

**Manuscript Number:**	PONE-D-25-23547
**Article Type:**	Research Article
**Full Title:**	Impact of Cilostazol on Clinical Outcomes in Lower Extremity Arterial Disease Patients After Angioplasty: A Real-World Analysis
**Short Title:**	Real-World Outcomes of Cilostazol in LEAD
**Corresponding Author:**	Yi-Heng Li<br>National Cheng Kung University Hospital<br>Tainan, TAIWAN
**Keywords:**	Cilostazol, lower extremity arterial disease, peripheral artery disease, angioplasty, antiplatelet therapy
**Abstract:**	Background<br>Cilostazol has been shown to improve walking distance in patients with lower extremity arterial disease (LEAD) and may reduce restenosis after revascularization. However, its long-term prognostic impact in real-world settings remains underexplored.<br>Methods<br>We conducted a retrospective cohort study using data from Taiwan's National Health Insurance Research Database (2012–2022). We included stable LEAD patients who had undergone percutaneous transluminal angioplasty (PTA) and remained event-free for 1 year. Stabilized inverse probability of treatment weighting (IPTW) was applied to adjust for baseline confounders. The study aimed to evaluate the effect of cilostazol on major adverse cardiovascular events (MACE), major adverse limb events (MALE), and composite bleeding outcomes.<br>Results<br>Among 5,300 stable LEAD patients, of whom 844 received cilostazol alone, 1,786 received aspirin or clopidogrel, and 2,670 received cilostazol combined with aspirin or clopidogrel. After IPTW, there were no significant differences between cilostazol monotherapy and any antiplatelet therapy groups regarding MACE, MALE, or composite bleeding outcomes (aHR [95% CI] = 0.84 [0.68–1.03], p=0.09; 0.84 [0.70–1.01], p=0.06; 0.88 [0.71–1.10], p=0.26, respectively). In secondary outcomes, cilostazol treatment was associated with a reduced rate of subsequent angioplasty (aHR [95% CI] = 0.80 [0.60–0.98], p = 0.03). There were no significant differences in clinical outcomes when comparing cilostazol monotherapy to cilostazol combined with antiplatelet therapy.<br>Conclusion<br>In this real-world Asian cohort, cilostazol showed similar prognostic benefits and safety compared to standard antiplatelet therapy. These findings support its role in the long-term management of LEAD patients following PTA, particularly in Asian populations.
**Order of Authors:**	Hsien-Yen Chang
	Hui-Wen Lin
	Po-Wei Chen
	Sheng-Hsiang Lin
	Ting-Hsing Chao
	Yi-Heng Li
**Additional Information:**	
**Question**	**Response**
**Financial Disclosure**	Yes

Powered by Editorial Manager® and ProduXion Manager® from Aries Systems Corporation

Enter a financial disclosure statement that describes the sources of funding for the work included in this submission. Review the submission guidelines for detailed requirements. View published research articles from *PLOS ONE* for specific examples.<br><br>This statement is required for submission and **will appear in the published article** if the submission is accepted. Please make sure it is accurate.<br><br>**Funded studies**<br>Enter a statement with the following details:<br>• Initials of the authors who received each award<br>• Grant numbers awarded to each author<br>• The full name of each funder<br>• URL of each funder website<br>• Did the sponsors or funders play any role in the study design, data collection and analysis, decision to publish, or preparation of the manuscript?<br><br>Did you receive funding for this work?	
Please add funding details.<br>as follow-up to "**Financial Disclosure**<br><br>Enter a financial disclosure statement that describes the sources of funding for the work included in this submission. Review the submission guidelines for detailed requirements. View published research articles from *PLOS ONE* for specific examples.<br><br>This statement is required for submission and **will appear in the published article** if the submission is accepted. Please make sure it is accurate.	This work was supported by a grant from the Chung Shan Medical University Hospital (CSH-2-25-D-002, Ting-Hsing Chao).

**Funded studies**<br>Enter a statement with the following details:<br>• Initials of the authors who received each award<br>• Grant numbers awarded to each author<br>• The full name of each funder<br>• URL of each funder website<br>• Did the sponsors or funders play any role in the study design, data collection and analysis, decision to publish, or preparation of the manuscript?<br><br>Did you receive funding for this work?"	
Please select the country of your main research funder (please select carefully as in some cases this is used in fee calculation).<br>as follow-up to "**Financial Disclosure**<br><br>Enter a financial disclosure statement that describes the sources of funding for the work included in this submission. Review the submission guidelines for detailed requirements. View published research articles from *PLOS ONE* for specific examples.<br><br>This statement is required for submission and **will appear in the published article** if the submission is accepted. Please make sure it is accurate.<br><br>**Funded studies**<br>Enter a statement with the following details:<br>• Initials of the authors who received each award<br>• Grant numbers awarded to each author<br>• The full name of each funder<br>• URL of each funder website<br>• Did the sponsors or funders play any role in the study design, data collection and analysis, decision to publish, or preparation of the manuscript?<br><br>Did you receive funding for this work?"	TAIWAN - TW
**Competing Interests**	The authors have declared that no competing interests exist.

Use the instructions below to enter a competing interest statement for this submission. On behalf of all authors, disclose any competing interests that could be perceived to bias this work—acknowledging all financial support and any other relevant financial or non-financial competing interests.<br><br>This statement is required for submission and **will appear in the published article** if the submission is accepted. Please make sure it is accurate and that any funding sources listed in your Funding Information later in the submission form are also declared in your Financial Disclosure statement.<br><br>View published research articles from *PLOS ONE* for specific examples.<br><br>**NO authors have competing interests**<br><br>Enter: *The authors have declared that no competing interests exist.*<br><br>**Authors with competing interests**<br><br>Enter competing interest details beginning with this statement:<br><br>*I have read the journal's policy and the authors of this manuscript have the following competing interests: [insert competing interests here]*<br><br>* typeset	
**Ethics Statement**<br><br>Enter an ethics statement for this submission. This statement is required if the study involved:<br><br>• Human participants<br>• Human specimens or tissue<br>• Vertebrate animals or cephalopods<br>• Vertebrate embryos or tissues	This study adhered to the Declaration of Helsinki and received approval from the Human Research and Ethics Committee of National Cheng Kung University Hospital (IRB number: A-EX-113-007)

Powered by Editorial Manager® and ProduXion Manager® from Aries Systems Corporation

• Field research<br><br>Write "N/A" if the submission does not require an ethics statement.<br><br>General guidance is provided below. Consult the submission guidelines for detailed instructions. **Make sure that all information entered here is included in the Methods section of the manuscript.**	

**Format for specific study types**<br><br>**Human Subject Research (involving human participants and/or tissue)**<br>• Give the name of the institutional review board or ethics committee that approved the study<br>• Include the approval number and/or a statement indicating approval of this research<br>• Indicate the form of consent obtained (written/oral) or the reason that consent was not obtained (e.g. the data were analyzed anonymously)<br><br>**Animal Research (involving vertebrate animals, embryos or tissues)**<br>• Provide the name of the Institutional Animal Care and Use Committee (IACUC) or other relevant ethics board that reviewed the study protocol, and indicate whether they approved this research or granted a formal waiver of ethical approval<br>• Include an approval number if one was obtained<br>• If the study involved *non-human primates*, add *additional details* about animal welfare and steps taken to ameliorate suffering<br>• If anesthesia, euthanasia, or any kind of animal sacrifice is part of the study, include briefly which substances and/or methods were applied<br><br>**Field Research**<br><br>Include the following details if this study involves the collection of plant, animal, or other materials from a natural setting:<br>• Field permit number<br>• Name of the institution or relevant body that granted permission	
**Data Availability**<br><br>Authors are required to make all data underlying the findings described fully available, without restriction, and from the time of publication. PLOS allows rare exceptions to address legal and ethical concerns. See the PLOS Data Policy and FAQ for detailed information.	No - some restrictions will apply

Powered by Editorial Manager® and ProduXion Manager® from Aries Systems Corporation

A Data Availability Statement describing where the data can be found is required at submission. Your answers to this question constitute the Data Availability Statement and **will be published in the article**, if accepted.<br><br>**Important:** Stating 'data available on request from the author' is not sufficient. If your data are only available upon request, select 'No' for the first question and explain your exceptional situation in the text box.<br><br>Do the authors confirm that all data underlying the findings described in their manuscript are fully available without restriction?	
**Describe where the data may be found in full sentences. If you are copying our sample text, replace any instances of XXX with the appropriate details.**<br><br>• If the data are **held or will be held in a public repository**, include URLs, accession numbers or DOIs. If this information will only be available after acceptance, indicate this by ticking the box below. For example: *All XXX files are available from the XXX database (accession number(s) XXX, XXX.*).<br>• If the data are all contained **within the manuscript and/or Supporting Information files**, enter the following: *All relevant data are within the manuscript and its Supporting Information files.*<br>• If neither of these applies but you are able to provide **details of access elsewhere**, with or without limitations, please do so. For example:<br><br>*Data cannot be shared publicly because of [XXX]. Data are available from the XXX Institutional Data Access / Ethics Committee (contact via XXX) for researchers who meet the criteria for access to confidential data.*<br><br>*The data underlying the results presented in the study are available from (include the name of the third party*	The datasets analyzed during the current study are derived from the National Health Insurance Research Database, Taiwan. Due to data protection regulations, the raw data are not publicly available.

and contact information or URL).<br>• This text is appropriate if the data are owned by a third party and authors do not have permission to share the data.<br><br>* typeset	
Additional data availability information:	

Powered by Editorial Manager® and ProduXion Manager® from Aries Systems Corporation

Manuscript Click here to access/download;Manuscript;Manuscript.docx

Impact of Cilostazol on Clinical Outcomes in Lower Extremity Arterial Disease Patients After Angioplasty: A Real-World Analysis

Hsien-Yuan Chang[1,2], Hui-Wen Lin[2], Po-Wei Chen[1,2], Sheng-Hsiang Lin[1,3,4], Ting-Hsing Chao[2,5*], Yi-Heng Li[2*].

1. Institute of Clinical Medicine, College of Medicine, National Cheng Kung University, Tainan, Taiwan
2. Division of Cardiology, Department of Internal Medicine, National Cheng Kung University Hospital, College of Medicine, National Cheng Kung University, Tainan, Taiwan
3. Biostatistics Consulting Center, National Cheng Kung University Hospital, College of Medicine, National Cheng Kung University, Tainan, Taiwan.
4. Department of Public Health, College of Medicine, National Cheng Kung University, Tainan, Taiwan.
5. Division of Cardiology, Department of Internal Medicine, Chung-Shan Medical University Hospital, School of Medicine, Chung Shan Medical University, Taichung, Taiwan

*These authors contributed equally to this work.

Corresponding author:

1. Dr. Yi-Heng Li

Department of Internal Medicine, National Cheng Kung University Hospital

138 Shengli Road, Tainan 704, Taiwan

TEL: +886-6- 2353535 ext.2389

FAX: +886-6-2753834

E-mail: heng@mail.ncku.edu.tw

2. Ting-Hsing Chao, MD

Department of Internal Medicine, National Cheng Kung University Hospital

138 Sheng-Li Road North District, Tainan 70403, Taiwan

TEL: +886-6-23523535 ext. 2392

FAX: +886-6-2753834

E-mail: chaotinghsing@gmail.com

Running Title: Real-World Outcomes of Cilostazol in LEAD

Conflict of interest declaration: All the authors declare no conflicts of interest.

Funding: This work was supported by a grant from the Chung Shan Medical University Hospital (CSH-2-25-D-002).

Ethical approval: This study adhered to the Declaration of Helsinki and received approval from the Human Research and Ethics Committee of National Cheng Kung University Hospital (IRB number: A-EX-113-007).

Statement of data access and integrity: The authors declare that they had full access to all the study data. The author(s) assume(s) complete responsibility for the integrity of the data and the accuracy of the data analysis.

Data availability statement: The datasets analyzed during the current study are derived from the National Health Insurance Research Database, Taiwan. Due to data protection regulations, the raw data are not publicly available.

Abstract

Background

Cilostazol has been shown to improve walking distance in patients with lower extremity arterial disease (LEAD) and may reduce restenosis after revascularization. However, its long-term prognostic impact in real-world settings remains underexplored.

Methods

We conducted a retrospective cohort study using data from Taiwan's National Health Insurance Research Database (2012–2022). We included stable LEAD patients who had undergone percutaneous transluminal angioplasty (PTA) and remained event-free for 1 year. Stabilized inverse probability of treatment weighting (IPTW) was applied to adjust for baseline confounders. The study aimed to evaluate the effect of cilostazol on major adverse cardiovascular events (MACE), major adverse limb events (MALE), and composite bleeding outcomes.

Results

Among 5,300 stable LEAD patients, of whom 844 received cilostazol alone, 1,786 received aspirin or clopidogrel, and 2,670 received cilostazol combined with aspirin or clopidogrel. After IPTW, there were no significant differences between cilostazol monotherapy and any antiplatelet therapy groups regarding MACE, MALE,

or composite bleeding outcomes (aHR [95% CI] = 0.84 [0.68–1.03], p=0.09; 0.84 [0.70–1.01], p=0.06; 0.88 [0.71–1.10], p=0.26, respectively). In secondary outcomes, cilostazol treatment was associated with a reduced rate of subsequent angioplasty (aHR [95% CI] = 0.80 [0.60–0.98], p = 0.03). There were no significant differences in clinical outcomes when comparing cilostazol monotherapy to cilostazol combined with antiplatelet therapy.

Conclusion

In this real-world Asian cohort, cilostazol showed similar prognostic benefits and safety compared to standard antiplatelet therapy. These findings support its role in the long-term management of LEAD patients following PTA, particularly in Asian populations.

Keywords: Cilostazol, lower extremity arterial disease, peripheral artery disease, angioplasty, antiplatelet therapy

Introduction

Lower extremity arterial disease (LEAD) ranks as the third leading cause of death among individuals with atherosclerotic cardiovascular diseases, following coronary artery disease and stroke. In recent years, advancements in endovascular revascularization have positioned it as a cornerstone in the management of symptomatic LEAD, especially for patients with intermittent claudication or critical limb ischemia. However, despite the initial technical success of these interventions, high rates of restenosis and recurrent symptoms remain prevalent.[1] These challenges underscore the importance of implementing guideline-directed medical therapy to optimize long-term clinical outcomes and maintain vascular patency following revascularization [2-4]

Cilostazol, a phosphodiesterase 3 inhibitor with antiplatelet and vasodilatory properties, is commonly used in LEAD patients to alleviate the symptoms of intermittent claudication and to reduce post-angioplasty restenosis. Several recent randomized controlled trials have demonstrated its efficacy in lowering late lumen loss and reducing the need for target lesion revascularization.[5] Beyond its vascular benefits, cilostazol has shown promising effects on angiogenesis in preclinical studies,[6-8] and emerging evidence suggests its potential in reducing major adverse cardiovascular events (MACE)[9,10]. However, despite these findings, current clinical

guidelines do not recommend cilostazol for the explicit purpose of MACE reduction,[2] and the European Society of Cardiology guidelines make no recommendation of cilostazol at all in the context of LEAD management.[4] Notably, although randomized controlled trials have highlighted the potential benefits of cilostazol, evidence from real-world clinical practice—particularly among LEAD patients following angioplasty—remains relatively limited. This discrepancy between trial-based findings and current clinical guideline recommendations invites further investigation into cilostazol's role in the management of LEAD in routine care settings.

Therefore, this study aims to compare cilostazol with other current antiplatelet therapies using real-world data, evaluating their impact on clinical outcomes, including MACE and major adverse limb events (MALE), in patients with LEAD undergoing endovascular revascularization in a real-world setting.

Methods

Study design

This is a retrospective cohort study using data from the National Health Insurance Research Database in Taiwan, which were accessed and processed for research purposes between November 1, 2024, and March 31, 2025, covering the period from January 1, 2012, to December 31, 2022. This study adhered to the Declaration of Helsinki and received approval from the Institutional Review Board of

the National Cheng Kung University Hospital (IRB number: A-EX-113-007). The requirement for informed consent was waived owing to the retrospective design of the study. All data were fully anonymized before access, and the authors did not have access to any information that could identify individual participants during or after data collection

Study population and inclusion criteria

We enrolled adult patients who were newly diagnosed with LEAD and underwent percutaneous transluminal angioplasty (PTA). Medical information was recorded using the International Classification of Diseases, Ninth and Tenth Revisions, Clinical Modification (ICD-9-CM and ICD-10-CM) codes. Patients diagnosed with LEAD were identified using the following codes: ICD-9-CM codes (443.9, 440.2, 440.3, 440.4) and ICD-10-CM codes (I65.9, I63.00, I63.10, I63.20, I63.29, I73.9, I70.2, I70.3, I70.4, I70.5, I70.6, I70.7, I70.8). Patients who underwent PTA were identified using the following codes: ICD-9-CM codes (38.08, 38.18, 38.38, 38.48, 38.58, 35.68, 38.88, 39.50, 39.7, 39.90, 39.25, 39.26, 39.29) and ICD-10-CM codes (041, 045, 047, 049, 04B, 04C, 04H, 04J, 04L, 04N, 04P, 04Q, 04R, 04S, 04U, 04V, 04W).

In line with current guidelines that recommend dual antiplatelet therapy (DAPT) for 1–3 months post-PTA,[2,4] To avoid immortal time bias, a landmark design was

ployed,[11] with the index date set between six months and one year after PTA. Clinical information collected during the index date included age, sex, comorbidities, and medications. Detailed corresponding ICD codes are listed in Supplemental Table 1. Patients were grouped according to the medications they were using at the index date. To focus the analysis on the long-term effects of the medications on clinical outcomes, patients who experienced major adverse cardiovascular events (MACE) within the first year following PTA or major adverse limb events (MALE) between six months and one year post-angioplasty were excluded from the study (Supplemental Figure 1). Additional exclusion criteria included medication records of fewer than 84 days within the index date, use of aspirin plus clopidogrel, previous history of atrial fibrillation, or other indications for novel oral anticoagulants or warfarin.

Clinical outcomes

Clinical outcomes were recorded starting from one year after PTA. Detailed corresponding ICD codes are listed in Supplemental Table 1. The primary endpoint was the incidence of MACE, defined as a composite outcome of cardiovascular death, non-fatal myocardial infarction, stroke, or transient ischemic attack. The co-primary endpoint was the incidence of MALE, defined as a composite outcome of undergoing PTA for LEAD or amputation. The secondary endpoints were cardiovascular death, non-fatal myocardial infarction, stroke or transient ischemic attack, undergoing PTA

for LEAD, and amputation. The safety outcome was a composite of bleeding events, including hemorrhagic stroke, gastrointestinal bleeding, or other site bleeding.

Statistical analysis

Continuous data are presented as mean ± standard deviation, while categorical data are expressed as frequencies and percentages. Comparisons among the three groups were conducted using one-way ANOVA for normally distributed continuous variables, and the Kruskal–Wallis test for non-normally distributed variables. For categorical variables, either the chi-square test or Fisher's exact test was applied, as appropriate. To minimize confounding due to baseline differences among groups, stabilized inverse probability of treatment weighting (IPTW) was applied. Propensity scores were estimated using a multinomial logistic regression model based on baseline covariates, including age, gender, times of PTA, comorbidities (hypertension, diabetes mellitus, dyslipidemia, coronary artery disease, chronic kidney disease, end stage renal disease), and medications (statin, beta blocker, etc.). Stabilized weights were calculated as the marginal probability of group assignment divided by the propensity score for each individual. These weights were used to construct a pseudopopulation in which the distribution of baseline covariates was balanced across the three groups. After applying IPTW, covariate balance among the three groups was assessed using one-way ANOVA or Kruskal–Wallis tests for continuous variables and

chi-square or Fisher's exact tests for categorical variables. These comparisons ensured that the weighted sample achieved adequate balance in baseline characteristics.

Cox proportional hazards models were used to estimate hazard ratios and 95% confidence intervals for outcomes across the groups. Both IPTW-weighted and unweighted models were constructed. In the unweighted models, multivariable adjustment for the aforementioned baseline covariates was performed. The proportional hazards assumption was verified prior to model fitting. A two-sided p-value < 0.05 was considered statistically significant. All statistical analyses were conducted using SAS version 9.4 (SAS Institute, Cary, NC, USA).

Results

Study population

A total of 17,993 patients diagnosed with LEAD and treated with PTA were retrospectively screened. Of these, 7,760 patients were excluded due to the occurrence of MACE within one year or MALE on the index date. After applying additional exclusion criteria, a final cohort of 5,300 patients was included in the analysis (mean [illegible]: 71 ± 12 years; 61% male). Among these patients, 1,786 (34%) received aspirin or clopidogrel monotherapy, 844 (16%) received cilostazol monotherapy, and 2,670 (50%) were treated with cilostazol in combination with aspirin or clopidogrel (Figure 1). The most notable baseline difference was the prevalence of coronary artery disease

(CAD), which was significantly lower in the cilostazol group compared to the others (29% vs. 52%, $p < 0.01$, Table 1). This finding is clinically reasonable, as current guidelines recommend aspirin or clopidogrel for patients with CAD, making them less likely to be prescribed cilostazol monotherapy. In addition, despite their now-recognized therapeutic potential in the management of LEAD, newer pharmacological agents such as rivaroxaban 2.5mg, sodium-glucose cotransporter 2 (SGLT2) inhibitors, and glucagon-like peptide-1 (GLP-1) receptor agonists were prescribed in only 1–3% of patients in this real-world dataset. This likely reflects the treatment patterns of the earlier period during which the data were collected, prior to the widespread adoption of these agents based on more recent clinical evidence.

To account for differences in baseline characteristics—such as sex, number of PTA procedures, comorbidities, and concomitant medications—we applied IPTW. After adjustment, there were no significant differences among the three treatment groups (Table 1).

Clinical outcomes

The mean follow-up time was 2.68 years. During this period, the overall incidence rates of MACE, MALE, and composite bleeding were 16.3%, 22.9%, and 13.8%, respectively. When comparing cilostazol monotherapy with aspirin or clopidogrel monotherapy, there were no statistically significant differences in the

incidence of MACE (adjusted hazard ratio (aHR), 0.84; 95% CI, 0.68–1.03; $p = 0.09$) or MALE (aHR, 0.84; 95% CI, 0.70–1.01; $p = 0.06$, Table 2. Among the secondary endpoints, cilostazol monotherapy was associated with a significantly lower risk of repeat PTA (aHR, 0.80; 95% CI, 0.66–0.98; $p = 0.03$). While there were no significant differences in the overall incidence of composite bleeding across groups, a near-significant reduction in gastrointestinal bleeding was observed in the cilostazol monotherapy group (aHR, 0.76; 95% CI, 0.57–1.01; $p = 0.05$). These findings suggest that cilostazol monotherapy may offer comparable clinical outcomes relative to aspirin or clopidogrel monotherapy in routine clinical practice.

In comparisons between aspirin or clopidogrel monotherapy and combination therapy with cilostazol, there were no statistically significant differences in the incidence of MACE (aHR, 0.87; 95% CI, 0.75–1.00; $p = 0.06$), MALE (aHR, 1.11; 95% CI, 0.97–1.26; $p = 0.12$), or composite bleeding (aHR, 0.94; 95% CI, 0.81–1.11; $p = 0.26$). Although the difference in MACE did not reach conventional statistical significance, the observed trend (95% CI, 0.75–1.00) suggests a possible directional benefit with the addition of cilostazol to standard antiplatelet therapy. While not statistically significant, the proximity of the estimate to the threshold may indicate potential clinical relevance.

Subgroup analyses

Subgroup analyses were performed based on key baseline characteristics, including age, sex, times of PTA, diabetes, hypertension, dyslipidemia, coronary artery disease, chronic kidney disease, and end stage renal disease (Figure 2 and Supplemental figure 2). Overall, the treatment effects were generally consistent across subgroups. The direction and magnitude of treatment effects remained similar in most strata, suggesting broadly consistent results across the study population.

Discussion

In this real-world cohort of stable LEAD patients who underwent PTA, cilostazol therapy was associated with clinical outcomes comparable to those observed with aspirin or clopidogrel therapy. No statistically significant differences were noted in the incidence of MACE, MALE, or composite bleeding. In secondary analyses, cilostazol was associated with a significantly lower rate of repeat angioplasty, suggesting a potential benefit in reducing restenosis or recurrent limb ischemia.

LEAD remains a condition with a notably high mortality rate, surpassing that CAD and stroke.[12,13] A large cohort study reported event rates for all-cause mortality and MACE in LEAD patients to be approximately 113 and 71 per 1,000 person-years, respectively. In our cohort of 17,993 patients undergoing PTA—likely representing a population with more advanced disease—we observed a 21% one-year mortality rate and a 22% incidence of MACE or MALE. These strikingly high complication rates

underscore the critical need for comprehensive care strategies that extend beyond revascularization alone. Early detection and the implementation of guideline-directed medical therapy are crucial cornerstones in managing LEAD patients. These findings further emphasize the importance of evaluating the potential benefits of individual pharmacologic therapies in improving outcomes in this high-risk population.

Cilostazol has been shown to exert multiple pleiotropic effects that may offer meaningful clinical benefits to patients with LEAD. Beyond its primary pharmacologic actions, inhibition of platelet aggregation and induction of vasodilation, cilostazol has demonstrated the ability to protect against apoptotic cell death,[14] anti-inflammation,[15] promote angiogenesis, and enhance endothelial function[6-8]. Clinically, these biological effects translate into improved patient outcomes, including reduction in claudication symptoms,[16] increase walking distance,[17] and a lower risk of restenosis, re-occlusion, target lesion revascularization,[18] and even amputation.[19] Our findings complement and expand upon previous evidence by showing, in a real-world cohort, that cilostazol monotherapy was associated with a significantly lower rate of repeat PTA procedures compared to aspirin or clopidogrel monotherapy, without increasing the risk of MACE or bleeding. Although real-world data are inherently prone to selection bias, it is worth noting that cilostazol is often prescribed to patients with more pronounced

symptoms. The observed reduction in MALE events may therefore reflect cilostazol's clinical effectiveness. Conversely, this same bias may partially explain why combination therapy with cilostazol and aspirin or clopidogrel did not show a statistically significant benefit over single-agent therapy, as those patients might have had more advanced disease at baseline.

In addition to its benefits in reducing MALE, cilostazol has also been evaluated for its potential role in lowering the risk of MACE.[20] A randomized controlled trial in high-risk cardiovascular patients reported a hazard ratio of 0.67 for MACE in the cilostazol group, suggesting a possible protective effect, particularly in subgroups with diabetes or prior percutaneous coronary intervention.[10] Similarly, a meta-analysis of 15 trials demonstrated a significant reduction in MACE risk (RR = 0.67; 95% CI, 0.56–0.81), although no difference in all-cause mortality was observed.[9] In hemodialysis patients with LEAD, cilostazol was associated with improved 10-year MACE-free survival (HR 0.57; 95% CI, 0.41–0.79).[21] In our real-world dataset, cilostazol monotherapy showed no significant difference in MACE risk compared to aspirin or clopidogrel. However, dual therapy combining cilostazol with either aspirin or clopidogrel yielded a hazard ratio for MACE (HR 0.87; 95% CI, 0.75–1.00) and stroke (HR 0.78; 95% CI, 0.60–1.00) that closely approached statistical significance. Subgroup analyses revealed generally consistent effects across various patient

characteristics, further supporting the potential cardiovascular benefits of cilostazol in selected populations.

Several limitations should be considered when interpreting our findings. First, although we employed robust statistical adjustments using IPTW, the potential for residual confounding due to unmeasured variables—such as Rutherford classification, lesion severity, ankle-brachial index, degree of calcification, and lifestyle factors—cannot be entirely excluded. Second, as with all observational studies, the possibility of other uncontrolled sources of bias and confounding remains, warranting cautious interpretation of the results. Third, the data reflect clinical practice patterns between 2013 and 2020, a period when the adoption of newer cardiometabolic therapies—such as SGLT2 inhibitors, GLP-1 receptor agonists, and low-dose rivaroxaban (2.5 mg)—was limited. Therefore, the generalizability of our findings to more contemporary treatment paradigms may be constrained.

In conclusion, this nationwide real-world analysis suggests that cilostazol monotherapy provides clinical outcomes and safety comparable to conventional antiplatelet therapy in stable LEAD patients following PTA. In addition, cilostazol was associated with a reduced need for repeat revascularization, and signals of potential benefit in cardiovascular and limb outcomes were observed. These findings support its consideration as a viable therapeutic option in routine practice. Further randomized

trials or prospective studies are warranted to confirm these results and clarify cilostazol's role within the broader context of modern LEAD management.

Table 1. The baseline characteristics of LEAD patients treated with before and after inverse probability of treatment weighting (IPTW)

		Inverse probability of treatment weighting							
		Before				After			
	Total	Aspirin or Plavix users	Cilostazol users	Aspirin+Cilostazol or Plavix+Cilostazol	P	Aspirin or Plavix users	Cilostazol users	Aspirin+Cilostazol or Plavix+Cilostazol	P
	N=5300	N= 1786	N= 844	N= 2670		N=pseudo data	N=pseudo data	N=pseudo data	
Age (mean ± SD)	71.21 ± 11.61	70.78 ± 11.43	72.21 ± 12.30	71.19 ± 11.49	0.34	71.13 ± 11.51	71.00 ± 12.01	71.23 ± 11.51	0.39
Gender (Male)	3251 (61.34)	1136 (63.61)	471 (55.81)	1644 (61.57)		61.47	62.12	61.42	0.94
Times of PTA					<0.01				0.89
1	4443 (83.83)	1554 (87.01)	729 (86.37)	2160 (80.90)		83.72	84.53	83.79	
2	746 (14.08)	207 (11.59)	100 (11.85)	439 (16.44)		14.15	12.95	14.18	
≥3	111 (2.09)	25 (1.40)	15 (1.78)	71 (2.66)		2.13	2.51	2.03	
Comorbidity									
Hypertension	4446 (83.89)	1518 (84.99)	685 (81.16)	2243 (84.01)	0.04	83.88	84.10	83.82	0.98
Diabetes mellitus	3791 (71.53)	1278 (71.56)	583 (69.08)	1930 (72.28)	0.20	71.25	71.10	71.92	0.85
Dyslipidemia	2270 (42.83)	776 (43.45)	322 (38.15)	1172 (43.90)	0.01	43.03	42.83	42.70	0.98
Coronary artery disease	2407 (45.42)	935 (52.35)	250 (29.62)	1222 (45.77)	<0.01	45.44	45.14	45.41	0.99
Chronic kidney disease	2439 (46.02)	797 (44.62)	400 (47.39)	1242 (46.52)	0.32	46.50	45.12	46.10	0.83
End stage renal disease	56 (1.06)	19 (1.06)	14 (1.66)	23 (0.86)	0.14	1.11	1.14	1.03	0.95
Medications									
*Rivaroxaban 2.5mg	11 (0.21)				-	-			-

Statin	2728 (51.47)	971 (54.37)	333 (39.45)	1424 (53.33)	<0.01	51.32	51.11	51.40	0.99
Calcium channel blocker	1415 (26.70)	483 (27.04)	222 (26.3)	710 (26.59)	0.91	27.43	26.62	26.45	0.77
Beta blocker	2287 (43.15)	752 (42.11)	312 (36.97)	1223 (45.81)	<0.01	43.25	43.66	43.20	0.98
ACEi/ARB	2677 (50.51)	995 (55.71)	368 (43.60)	1314 (49.21)	<0.01	50.65	50.46	50.46	0.99
SGLT2 inhibitor	187 (3.53)	82 (4.59)	18 (2.13)	87 (3.26)	<0.01	3.60	3.41	3.58	0.98
GLP-1RA	69 (1.30)	21 (1.18)	11 (1.30)	37 (1.39)	0.83	1.12	1.61	1.39	0.60

Abbreviations: ACEi: Angiotensin-Converting Enzyme Inhibitor; ARB: Angiotensin II Receptor Blocker; GLP-1RA: Glucagon-Like Peptide-1 Receptor Agonist; PTA: Percutaneous Transluminal Angioplasty; SGLT2 Inhibitor: Sodium-Glucose Cotransporter 2 Inhibitor.

*Because the case number of thrombocytopenia was too small in one of the groups, the distribution of case number between groups was not allowed to be presented by the Taiwan Ministry of Health and Welfare.

Table 2. Outcomes of LEAD patients received cilostazol and/or antiplatelet

	Total (N=5300)	Aspirin or Plavix users . *(ref.)* (N=1786)	Cilostazol users (N=844)	Aspirin+Cilostazol or Plavix+Cilostazol (N=2670)		*Non IPTW		IPTW	
						Adjusted HR (95% CI)	p value	HR (95% CI)	p value
Death	1814 (34.23)	602 (33.71)	312 (36.97)	900 (33.71)	C	0.96 (0.84-1.10)	0.56	0.88 (0.76-1.01)	0.07
					A+P	0.91 (0.82-1.01)	0.08	0.90 (0.81-0.99)	0.04
MACEs	863 (16.28)	302 (16.91)	138 (16.35)	423 (15.84)	C	0.90 (0.73-1.10)	0.30	0.84 (0.68-1.03)	0.09
					A+P	0.86 (0.74-1.00)	0.05	0.87 (0.75-1.00)	0.06
Cardiovascular death	377 (7.11)	127 (7.11)	68 (8.06)	182 (6.82)	C	1.00 (0.74-1.35)	0.98	0.89 (0.65-1.20)	0.43
					A+P	0.87 (0.70-1.10)	0.25	0.87 (0.70-1.09)	0.22
Non-fatal MI	382 (7.21)	141 (7.89)	55 (6.52)	186 (6.97)	C	0.83 (0.60-1.14)	0.25	0.80 (0.58-1.09)	0.15
					A+P	0.82 (0.66-1.03)	0.08	0.85 (0.68-1.06)	0.14
Ischemic stroke/TIA	275 (5.19)	104 (5.82)	38 (4.50)	133 (4.98)	C	0.72 (0.49-1.05)	0.09	0.74 (0.51-1.06)	0.10
					A+P	0.81 (0.62-1.04)	0.10	0.78 (0.60-1.00)	0.05
MALEs	1213 (22.89)	383 (21.44)	163 (19.31)	667 (24.98)	C	0.84 (0.70-1.02)	0.07	0.84 (0.70-1.01)	0.06
					A+P	1.10 (0.97-1.25)	0.13	1.08 (0.96-1.23)	0.21
Angioplasty for LEAD	1087 (20.51)	342 (19.15)	136 (16.11)	609 (22.81)	C	0.80 (0.65-0.98)	0.03	0.80 (0.66-0.98)	0.03
					A+P	1.14 (0.99-1.30)	0.06	1.11 (0.97-1.26)	0.12
Amputation	325 (6.13)	108 (6.05)	53 (6.28)	164 (6.14)	C	0.95 (0.68-1.32)	0.75	0.89 (0.64-1.25)	0.50
					A+P	0.95 (0.74-1.21)	0.66	0.96 (0.75-1.21)	0.71
Composite bleeding	732 (13.81)	240 (13.44)	115 (13.63)	377 (14.12)	C	0.90 (0.72-1.13)	0.36	0.88 (0.71-1.10)	0.26

					A+P	0.96 (0.81-1.13)	0.61	0.94 (0.81-1.11)	0.48
Hemorrhagic stroke	45 (0.85)	17 (0.95)	6 (0.71)	22 (0.82)	C	0.66 (0.25-1.69)	0.38	0.60 (0.24-1.53)	0.28
					A+P	0.83 (0.44-1.57)	0.57	0.77 (0.42-1.42)	0.40
Gastrointestinal bleeding	470 (8.87)	160 (8.96)	65 (7.7)	245 (9.18)	C	0.76 (0.57-1.02)	0.07	0.76 (0.57-1.01)	0.05
					A+P	0.93 (0.76-1.14)	0.47	0.91 (0.75-1.10)	0.33
Other site bleeding	331 (6.25)	101 (5.66)	62 (7.35)	168 (6.29)	C	1.16 (0.84-1.61)	0.36	1.12 (0.81-1.53)	0.50
					A+P	1.02 (0.79-1.31)	0.89	1.00 (0.79-1.28)	0.99

Abbreviations: LEAD: CI, confidence interval; HR, hazard ratio; Lower Extremity Arterial Disease; MACE: Major Adverse Cardiovascular Events; MALE: Major Adverse Limb Events; MI: Myocardial Infarction; TIA: Transient Ischemic Attack.

* This model represents the pre-IPTW analysis, with adjustment for age, gender, times of PTA, comorbidity (hypertension, dyslipidemia, coronary artery disease), and medications (statin, beta blocker, ACEi/ARB, SGLT2 inhibitor).

Supplemental table 1. ICD-9 and ICD-10 codes

Disease	ICD-9 Codes	ICD-10 Codes
Peripheral artery disease	443.9, 440.2, 440.3, 440.4	I65.9, I63.00, I63.10, I63.20, I63.29, I73.9, I70.2, I70.3, I70.4, I70.5, I70.6, I70.7, I70.8
Comorbidities		
Hypertension	401,402,403,404,405	I10-I16
Diabetes mellitus	250	E10, E11
Hyperlipidemia	272	E78
Chronic kidney disease	580-589	I12, I13, N00, N01, N02, N03, N04, N05, N07, N11, N14, N17, N18, N19, Q61
ESRD	585.6	N18.6; Z99.2
Congestive heart failure	428	I5020, I5021, I5022, I5023, I5030<br>I5031, I5032, I5033, I5040, I5041<br>I5042, I5043, I509, I501
Atrial fibrillation	427.31	I48.0, I48.1, I48.2, I48.9
Coronary artery disease	410,411,412,413,414	I21-I25
Ischemic stroke	433, 434	I63
Transient ischemic attack	435.9	G45.9
Hemorrhagic stroke	430-432	I61
Outcome		
MACE		
Ischemic stroke	433, 434	I63
Transient ischemic attack	435.9	G45.9
Hemorrhagic stroke	430-432	I61
Non-fatal MI	410	I21,I22,I23
Safety outcome		
Hemorrhagic stroke	430-432	I61
Gastrointestinal bleeding	456.0, 456.2, 455.2, 455.5, 455.8, 530.7, 530.82,	K22.6, K25.0, K25.2, K25.4, K25.6, K26.0,

	531.0–531.6, 532.0– 532.6, 533.0–533.6, 534.0–534.6, 535.0– 535.6 537.83, 562.02, 562.03, 562.12 562.13 568.81, 569.3, 569.85, 578.0, 578.1, 578.9	K26.2, K26.4, K26.6, K27.0, K27.2, K27.4, K27.6, K28.0, K28.2, K28.4, K28.6, K29.0, K62.5, K92.0, K92.1, K92.2, I85.01
Other critical sites bleeding	423,0, 459.0, 568.81, 593.81, 599.7, 623.8, 626.32, 626.6, 719.1, 784.7, 784.8, 786.3, 519.09, 785.59, 958.4, 860.2, 860.3, 861.21, 861.31, 862.0, 862.1, 862.21, 862.31, 862.22, 901.0, 901.40, 901.41, 901.42, 864.00, 864.10, 965.00, 902.9, 865.10	D62, J942, H113, H356, H431, N02, N95, R04, R31, R58, I71, K66.1, J95.01, R57.1, J94.2, T79.4XXA, S27.1XXA, S27.329A, S26.01XA, S27.809A, S27.409A, S27.819A, S25.00XA, S25.409A, S36.119A, S36.00XA, S35.90XA, S36.90XA, S35.00XA, S35.10XA, S35.299A, S35.403A, S35.513A, S31.001A, T79.4XXA, S37.009A, S37.90XA, S48.019A, S48.919A, S58.019A, S58.919A, S68.419A, S78.019A, S78.919A, S88.019A, S88.119A, S98.019A
Procedures		
PTA	38.08, 38.18, 38.38, 38.48, 38.58, 35.68, 38.88, 39.50, 39.7, 39.90, 39.25, 39.26, 39.29	041, 045, 047, 049, 04B, 04C,<br>04H, 04J, 04L, 04N, 04P, 04Q, 04R, 04S, 04U, 04V, 04W
Amputation	84	0Y6

Figure legends.

Figure 1. Flow diagram of the study cohort. (MACE: major adverse cardiovascular events; MALE: major adverse limb events; N: number of participants; NOAC: Novel oral anticoagulants; PTA: percutaneous transluminal angioplasty)

Figure 2. Subgroup analysis of major adverse cardiovascular events and major adverse limb events between cilostazol users and aspirin or Plavix users.

Supplemental Figure 1. Schematic diagram of the data collection timeline. (LEAD: lower extremities artery disease; MACE: major adverse cardiovascular events; MALE: major adverse limb events; N: number of participants; NOAC: Novel oral anticoagulants; PTA: percutaneous transluminal angioplasty)

Supplemental Figure 2. Subgroup analysis of major adverse cardiovascular events and major adverse limb events between cilostazol plus aspirin or Plavix users and aspirin or Plavix users.

References

1. Callegari S, Romain G, Capuano I, et al. Association between guideline-directed medical therapy and reintervention risk following peripheral vascular interventions in patients with peripheral artery disease. Vascular medicine (London, England) 2025:1358863x251320347.

2. Gornik HL, Aronow HD, Goodney PP, et al. 2024 ACC/AHA/AACVPR/APMA/ABC/SCAI/SVM/SVN/SVS/SIR/VESS Guideline for the Management of Lower Extremity Peripheral Artery Disease: A Report of the American College of Cardiology/American Heart Association Joint Committee on Clinical Practice Guidelines. Circulation 2024;149:e1313-e410.

3. Criqui MH, Matsushita K, Aboyans V, et al. Lower Extremity Peripheral Artery Disease: Contemporary Epidemiology, Management Gaps, and Future Directions: A Scientific Statement From the American Heart Association. Circulation 2021;144:e171-e91.

4. Mazzolai L, Teixido-Tura G, Lanzi S, et al. 2024 ESC Guidelines for the management of peripheral arterial and aortic diseases. European heart journal 2024;45:3538-700.

5. Cerecedo CD, Silva A, Alia VS, et al. Cilostazol after endovascular therapy for femoropopliteal peripheral arterial disease: A systematic review and meta-analysis. Journal of vascular surgery 2025;81:767-74.e7.

6. Tseng SY, Chang HY, Li YH, Chao TH. Effects of Cilostazol on Angiogenesis in Diabetes through Adiponectin/Adiponectin Receptors/Sirtuin1 Signaling Pathway. International journal of molecular sciences 2022;23.

7. Tseng SY, Chao TH, Li YH, et al. Cilostazol improves high glucose-induced impaired angiogenesis in human endothelial progenitor cells and vascular endothelial cells as well as enhances vasculoangiogenesis in hyperglycemic mice mediated by the adenosine monophosphate-activated protein kinase pathway. Journal of vascular surgery 2016;63:1051-62.e3.

8. Chao TH, Tseng SY, Chen IC, et al. Cilostazol enhances mobilization and proliferation of endothelial progenitor cells and collateral formation by modifying vasculo-angiogenic biomarkers in peripheral arterial disease. International journal of cardiology 2014;172:e371-4.

9. Tan CH, Wu AG, Sia CH, et al. Cilostazol for secondary stroke prevention: systematic review and meta-analysis. Stroke and vascular neurology 2021;6:410-23.

10. Lin JL, Tseng WK, Lee PT, et al. A Randomized Controlled Trial Evaluating Outcome Impact of Cilostazol in Patients with Coronary Artery Disease or at a High Risk of Cardiovascular Disease. Journal of personalized medicine 2022;12.

11. Morgan CJ. Landmark analysis: A primer. Journal of nuclear cardiology : official publication of the American Society of Nuclear Cardiology 2019;26:391-3.

12. Hirsch AT, Hartman L, Town RJ, Virnig BA. National health care costs of peripheral arterial disease in the Medicare population. Vascular medicine (London, England) 2008;13:209-15.
13. Agnelli G, Belch JJF, Baumgartner I, Giovas P, Hoffmann U. Morbidity and mortality associated with atherosclerotic peripheral artery disease: A systematic review. Atherosclerosis 2020;293:94-100.
14. Lim JH, Woo JS, Shin YW. Cilostazol protects endothelial cells against lipopolysaccharide-induced apoptosis through ERK1/2- and P38 MAPK-dependent pathways. The Korean journal of internal medicine 2009;24:113-22.
15. Sakamoto T, Ohashi W, Tomita K, Hattori K, Matsuda N, Hattori Y. Anti-inflammatory properties of cilostazol: Its interruption of DNA binding activity of NF-κB from the Toll-like receptor signaling pathways. International immunopharmacology 2018;62:120-31.
16. Dawson DL, Cutler BS, Meissner MH, Strandness DE, Jr. Cilostazol has beneficial effects in treatment of intermittent claudication: results from a multicenter, randomized, prospective, double-blind trial. Circulation 1998;98:678-86.
17. Collinson DJ, Donnelly R. Cilostazol: improving walking distance in patients with intermittent claudication. Expert review of cardiovascular therapy 2004;2:503-9.
18. Soga Y, Iida O, Kawasaki D, Hirano K, Yamaoka T, Suzuki K. Impact of cilostazol on angiographic restenosis after balloon angioplasty for infrapopliteal artery disease in patients with critical limb ischemia. European journal of vascular and endovascular surgery : the official journal of the European Society for Vascular Surgery 2012;44:577-81.
19. Neel JD, Kruse RL, Dombrovskiy VY, Vogel TR. Cilostazol and freedom from amputation after lower extremity revascularization. Journal of vascular surgery 2015;61:960-4.
20. Sohn M, Chun EJ, Lim S. Cilostazol treatment for preventing adverse cardiovascular events in patients with type 2 diabetes and coronary atherosclerosis: Long-term follow-up of the ESCAPE study. Journal of diabetes 2022;14:524-31.
21. Ishii H, Aoyama T, Takahashi H, et al. Treatment with cilostazol improves clinical outcome after endovascular therapy in hemodialysis patients with peripheral artery disease. Journal of cardiology 2016;67:199-204.

Figure 1

Click here to access/download;Figure;Figure 1.tif

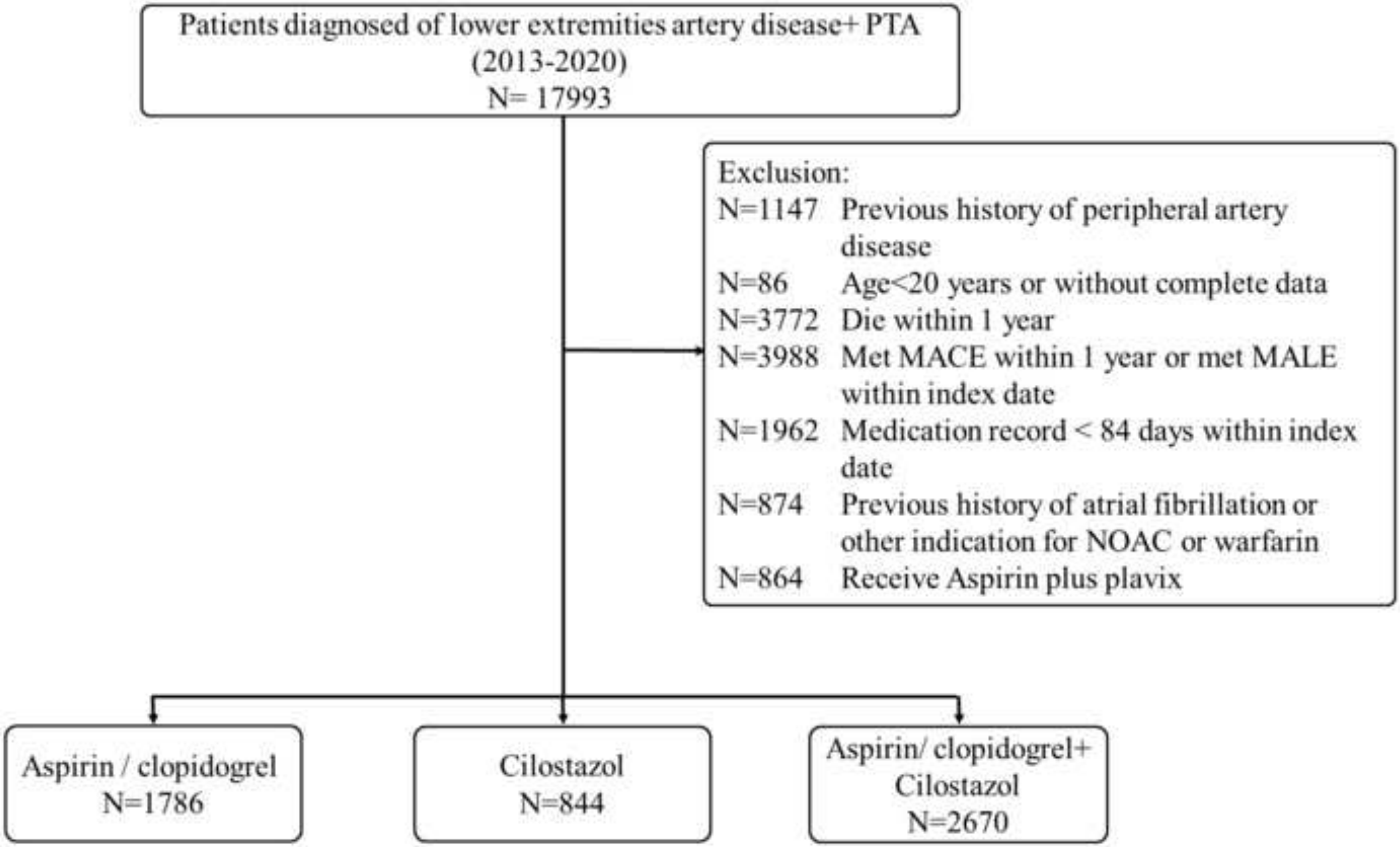

Figure 2

Click here to access/download;Figure;Figure 2.tif

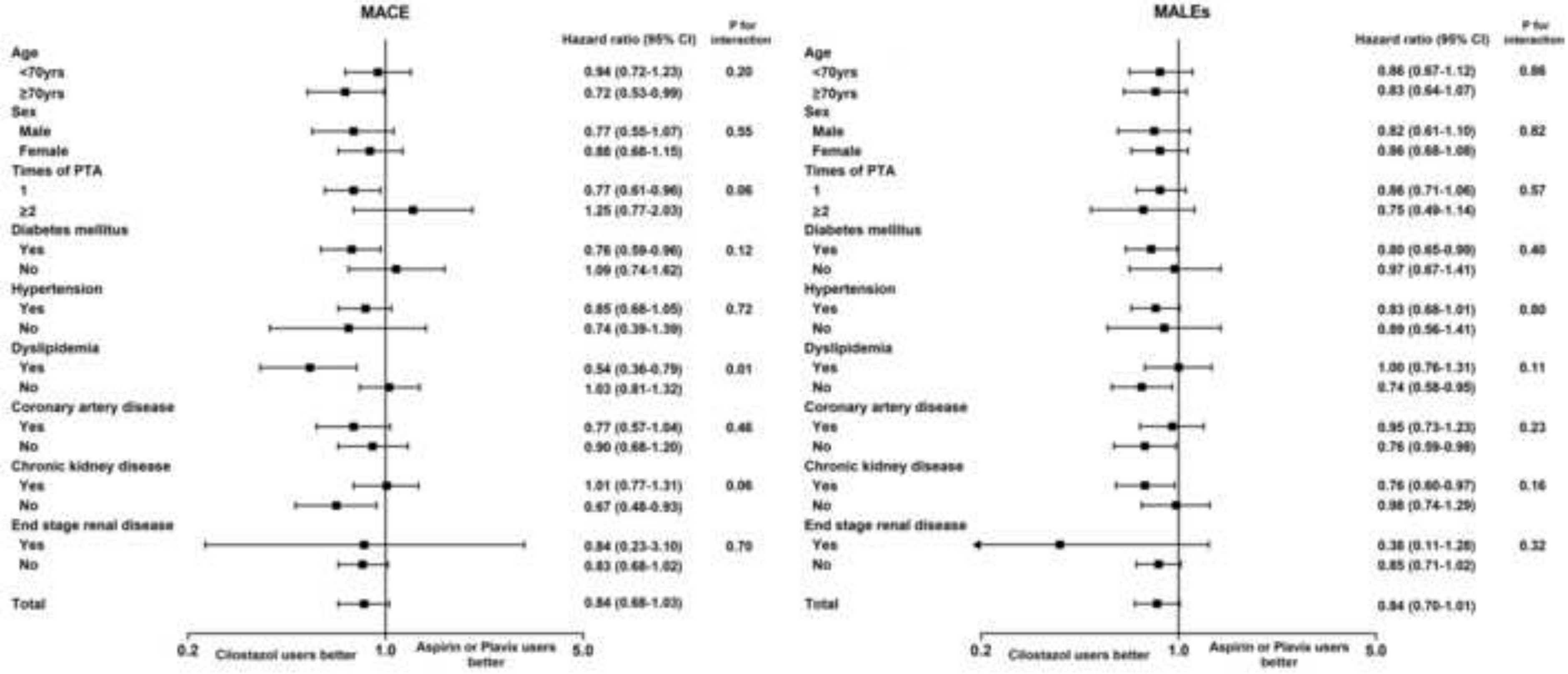

Supplemental figure 1

Click here to access/download
Supporting Information
Supplemental figure 1.tif

Supplemental figure 2

Click here to access/download
Supporting Information
Supplemental figure 2.tif
