# Supplementary material for: Impact of cilostazol on clinical outcomes in lower extremity arterial disease patients after angioplasty: A real-world analysis
Source: PLoS One. 2025 Aug 21;20(8):e0330434. doi: 10.1371/journal.pone.0330434 (PMC12370041; doi:10.1371/journal.pone.0330434)
Supplement: Attachment — Submitted filename: PLOS one Cilostazole peer review (1).docx [file pone.0330434.s005.docx]

Re: Impact of Cilostazol on Clinical Outcomes in Lower Extremity Arterial Disease Patients After Angioplasty: A Real-World Analysis

General Comments

This study significantly contributes to understanding cilostazol's long-term effects in LEAD patients post-PTA in a real-world setting. Its strengths lie in using Taiwan's National Health Insurance Research Database (NHIRD), providing a comprehensive and representative view of clinical practice over ten years. The large sample size of 5,300 stable LEAD patients enhances statistical power and allows for robust analysis. The robust statistical methodology, including stabilized Inverse Probability of Treatment Weighting (IPTW) for confounder adjustment and a landmark design to mitigate immortal time bias, significantly strengthens internal validity.

Despite these strengths, inherent limitations exist. As an observational study, there is potential for residual confounding from unmeasured variables (e.g., Rutherford classification, lesion severity, lifestyle factors) not captured in the administrative database. The generalizability is constrained by its reliance on an Asian cohort and a data collection timeframe (2012-2022) that predates widespread adoption of newer cardiometabolic therapies, limiting applicability to contemporary clinical settings. Finally, the interpretation of statistically non-significant findings (e.g., p-values near 0.05) as "potential clinical relevance" requires more cautious phrasing, as it may over-interpret the evidence

Specific Comments

3.1. Title and Abstract

The title is precise, and the abstract is well-structured, summarizing key aspects and findings.

3.2. Introduction

The introduction effectively sets the clinical context, highlights the research gap, and clearly states the study's objective.

3.3. Methods

3.3.1. Study Design and Data Source

A retrospective cohort design utilized Taiwan's NHIRD (2012-2022). The 10-year period allows for long-term outcome evaluation, but evolving medical guidelines may limit contemporary generalizability.

3.3.2. Study Population and Inclusion/Exclusion Criteria

Adult LEAD patients post-PTA were included, identified by ICD codes. A landmark design (6-12 months post-PTA) mitigated immortal time bias. Exclusion of patients with early MACE/MALE focused on a stable cohort, improving internal validity for this subgroup but potentially limiting broader generalizability.

3.3.3. Clinical Outcomes

Primary endpoints were MACE and MALE; secondary endpoints included individual components and a composite bleeding safety outcome, all clearly defined.

3.3.4. Statistical Analysis

Stabilized IPTW successfully adjusted for baseline confounders, achieving covariate balance. Cox proportional hazards models were used, with the proportional hazards assumption verified. Potential residual confounding from unmeasured variables is acknowledged.

3.4. Results

Cilostazol monotherapy significantly reduced repeat PTA (aHR 0.80, p=0.03). A near-significant reduction in GI bleeding was also observed (aHR 0.76, p=0.05). The interpretation of p-values near 0.05 as "near-significant" or "possible directional benefit" is an over-interpretation and should be rephrased. Subgroup analyses generally showed consistent effects.

3.5. Discussion

Cilostazol therapy showed comparable outcomes and safety to conventional antiplatelet therapy, with a significant reduction in repeat angioplasty. High MACE/MALE rates in LEAD patients underscore the need for comprehensive care. Cilostazol's pleiotropic effects are discussed as potential mechanisms.

Limitations are transparently acknowledged: residual confounding from unmeasured variables, general observational study biases, and the data timeframe (2013-2020) limiting generalizability to contemporary practice due to limited adoption of newer therapies. The authors explicitly limit generalizability to Asian populations. Further studies are called for.

3.6. Conclusion

Cilostazol monotherapy provides comparable clinical outcomes and safety to conventional antiplatelet therapy in stable LEAD patients post-PTA, and was associated with reduced repeat revascularization. The phrasing "signals of potential benefit" for non-significant trends should be rephrased more cautiously.
